# Evaluation of morphological characteristics for incomplete discoid medial meniscus with an oversized posterior segment

**DOI:** 10.1186/s13018-022-03132-2

**Published:** 2022-04-20

**Authors:** Shotaro Araki, Masanori Tsubosaka, Hirotsugu Muratsu, Takao Inokuchi, Hiroaki Maruo, Hidetoshi Miya, Ryosuke Kuroda, Takehiko Matsushita

**Affiliations:** 1 Department of Orthopaedic Surgery, Steel Memorial Hirohata Hospital, 3-1 Yumesaki-chou Hirohata-ku, Himeji-City, Hyogo 671-1122 Japan; 2 Department of Orthopaedic Surgery, Kobe University Graduate School of Medicine, Kobe, Japan

**Keywords:** Medial meniscus, Discoid, Oversize posterior segment, Morphological characteristic

## Abstract

**Background:**

A discoid medial meniscus is rare in comparison with a discoid lateral meniscus. We encountered a new type of incomplete discoid with an oversized posterior segment. Therefore, this study aimed to report cases of medial meniscus with an oversized posterior segment and analyze the morphological characteristics by comparing them to cases with a discoid medial meniscus and normal medial meniscus.

**Methods:**

Four patients with an oversized posterior segment medial meniscus (oversize group, mean age: 25.3 ± 12.0 years) and seven patients with a discoid medial meniscus (discoid group, mean age: 34.4 ± 19.6) were identified using magnetic resonance imaging (MRI) and diagnosed by arthroscopic findings in our hospital. Fifty patients without medial meniscal injury were retrospectively selected as the normal group (normal group, mean age: 24.0 ± 11.3 years). The clinical symptoms were examined. The anteroposterior (AP) length of both the anterior and posterior segments, AP length ratio of the posterior segment to the AP length of the medial tibial plateau, and mediolateral (ML) width of the mid-body of the medial meniscus were also evaluated using MRI and compared among the three groups.

**Results:**

All patients in the oversize group complained of medial knee pain during deep knee flexion. In sagittal MRI, posteriorly deviated indentations were also observed at the medial tibial plateau in all cases in the oversize group. There was a significant difference in the AP length of the posterior segment between the normal and oversize groups (14.3 ± 2.8 vs. 23.6 ± 2.8 mm, *P* < 0.001), whereas there was no significant difference in the AP length of the anterior segment (9.1 ± 2.1 vs. 9.5 ± 1.9 mm, *P* = 0.869). The ML width of the mid-body in the normal, oversize, and discoid groups was 9.3 ± 1.8, 19.9 ± 2.6, and 25.8 ± 1.9 mm, respectively (normal vs. oversize group: *P* < 0.001, oversize vs discoid group: *P* = 0.01, normal vs. discoid group: *P* < 0.001).

**Conclusions:**

Oversized posterior and normal anterior segments characterize this new type of incomplete discoid medial meniscus as a morphological abnormality.

## Background

The discoid medial meniscus, first reported by Cave and Staples in 1941 [1], is exceedingly rare compared to the discoid lateral meniscus [2–5]. The incidence of the discoid medial meniscus is as low as 0.12% [6], whereas that of the discoid lateral meniscus ranges from 0.4% to 17% [7–13].

Discoid meniscal tears occur in relatively younger patients. Generally, discoid medial meniscus symptoms often appear according to the symptoms of meniscal tears, and the symptoms do not occur only in the presence of the discoid meniscus [14]. The clinical symptoms of discoid medial meniscus include swelling, pain in the knee joint, and clicking. The symptoms, although relatively mild, worsen due to the progression of meniscal injury. Therefore, the discoid medial meniscus is often discovered after the meniscus is injured, and these patients are surgically treated with arthroscopic discoid meniscectomy [15–17].

The complete type, which covers the entire articular surface of the medial tibial plateau, has been described in previous reports on the discoid medial meniscus [3–5, 18]. A complete discoid meniscus can be diagnosed on sagittal magnetic resonance imaging (MRI), showing continuity between the anterior and posterior horns of the meniscus in three consecutive cuts. Kocher et al. reported that further confirmation could be obtained with the coronal images showing a transverse meniscal diameter > 15 mm or an involvement of > 20% of the tibial width [19]. However, the incomplete medial discoid type has not been mentioned in previous reports.

We encountered four rare cases of a medial meniscus with a larger posterior segment than the normal medial meniscus, but normal anterior segment. The purpose of this study was to report “this subtype discoid medial meniscu**s**” including case reports and analyze the morphological characteristics in comparison with discoid medial menisci and normal menisci.

## Methods

This study was approved by the Institutional Ethics Review Committee. Between 2006 and 2018, four patients with an oversized posterior segment medial meniscus (oversize group, mean age: 25.3 ± 12.0 years, three men and one woman) and seven patients with discoid medial meniscus (discoid group, mean age: 34.4 ± 19.6 years, five men and two women) were identified using magnetic resonance imaging (MRI) and diagnosed by arthroscopic findings in our hospital. A total of 50 patients with an isolated anterior cruciate ligament injury without medial meniscus injury were retrospectively selected as the normal group between 2015 and 2018 (normal group, mean age: 24.0 ± 11.3 years, 25 men and 25 women).

Standing anteroposterior and lateral radiographs of the knee were obtained for all the patients on their first visit to our hospital. The meniscal morphology and type of tear were evaluated with MRI using fat suppression T2 weighted imaging. The axial magnetic resonance images were sliced perpendicular to the tibial axis; coronal images were sliced parallel to the connecting line between the medial and lateral condyles of the femur; and sagittal images were sliced perpendicular to the line between the connecting line between the medial and lateral condyles of the femur. The slice width on MRI was 3 mm.

### MRI measurements

**Fig. 1:**
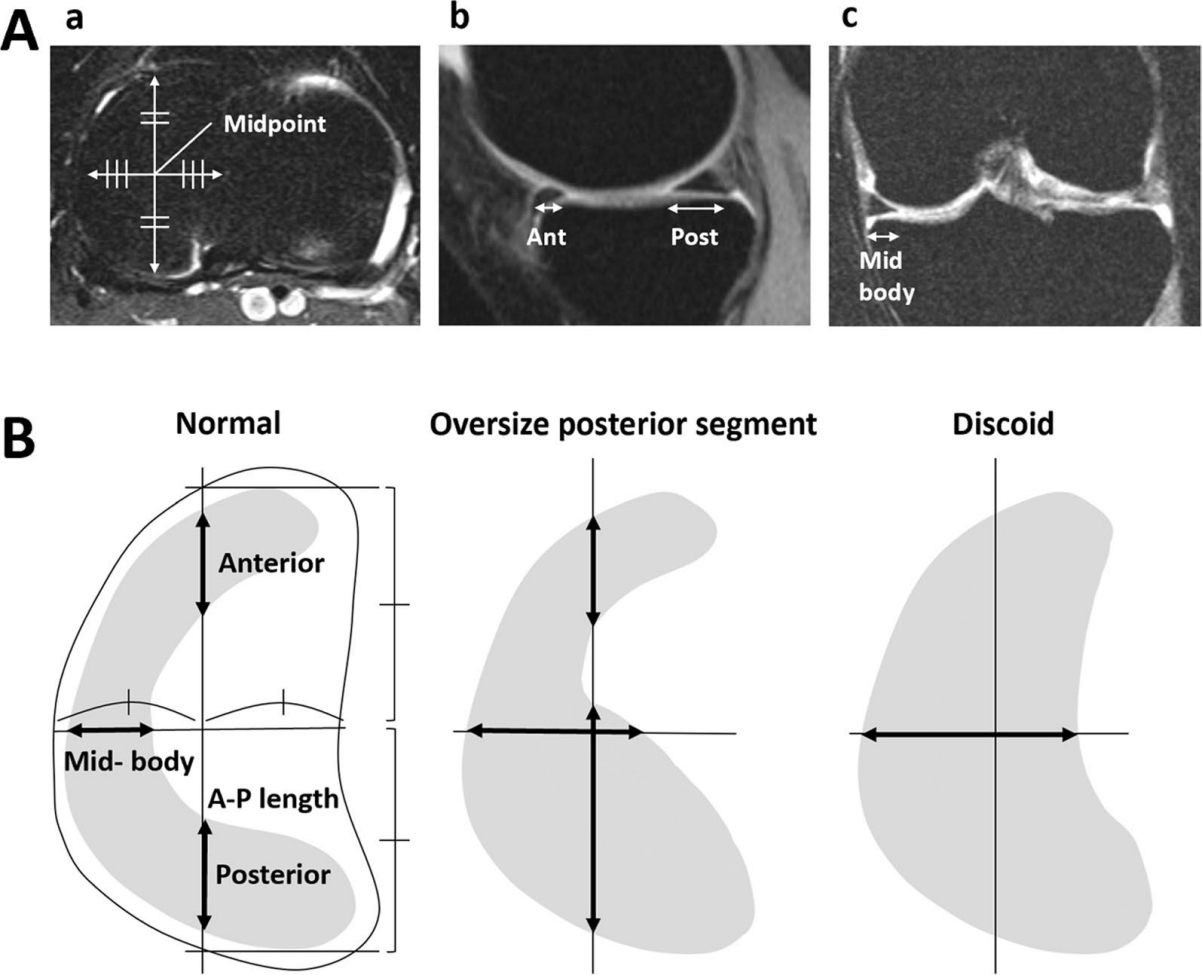
**A** Measurement methods in magnetic resonance imaging. (a) An axial view at the medial meniscus level. (b) The anteroposterior (AP) length of the medial meniscus is measured on sagittal-view images sectioning the midpoint between the medial edge of the tibia and the medial intercondylar eminence. (c) The mediolateral (ML) width of the medial meniscus is measured on the coronal-view images sectioning the midpoint of the AP width of the medial tibial plateau. **B** Illustrations showing the measurements in the normal, oversize, and discoid groups

The anteroposterior (AP) lengths of the anterior and posterior segments of the medial meniscus were measured using the sagittal image at the midpoint of the medial tibial plateau between the medial edge and tip of the medial intercondylar eminence (Fig. 1A ab, B). The mediolateral (ML) width of the mid-body was measured on the coronal image, which was sectioned at the midpoint of the AP length of the medial tibial plateau (Fig. 1A ac, B). The AP length ratio of the posterior segment relative to the medial tibial plateau was calculated. If the AP length of the posterior segment of the medial meniscus exceeded 19.9 mm, we named the menisci with large posterior segments as “oversize posterior segment medial meniscus.”

In the discoid group, only the ML width of the mid-body was measurable because we could not identify both the anterior and posterior segments (Fig. 1B). The AP lengths of the anterior and posterior segments were compared between the oversize and normal groups. The ML widths of the mid-body were compared between the oversize, discoid, and normal groups.

### Statistical analysis

Statistical analyses were performed using StatView software 5.0 (Abacus Concepts, Berkeley, CA, USA). Comparisons between the two groups were made using the Mann–Whitney U test. The Kruskal–Wallis test was used for comparison among the three groups, and the Bonferroni–Dunn method was used for post hoc analysis. All the tests were two-tailed with a confidence level of 95% (*P* < 0.05). The values are expressed as the mean ± standard deviation.

## Results

The demographic data of the three groups are shown in Table 1. All the patients, including those in the oversize and discoid groups, experienced pain in the knee joint. However, especially in the oversize group, pain with deep knee flexion was observed in all patients on physical examination. Conversely, three patients (42.9%) in the discoid group complained of deep flexion pain. Catching/clicking was observed in approximately 50% of the patients in both groups.

**Table 1:** Demographic data

	Normal	Oversize	Discoid	*P* value
Gender				
Male	25	3	5	–
Female	25	1	2	
Mean age (years) (range)	24.1^a^ (8–54)	25.3^b^ (14–38)	34.4^c^ (12–68)	^a, b^, *P* = 0.933 ^b, c^, *P* = 0.352 ^c, a^, *P* = 0.275
BMI (range)	23.0^a^ (17.7–33.5)	19.6^b^ (17.6–21.4)	21.7^c^ (15.0–26.0)	^a, b^, *P* = 0.023 ^b, c^, *P* = 0.171 ^c, a^, *P* = 0.801
Pain with deep knee flexion	–	4/4	3/7	–
Catching and click	–	2/4	4/7	–
Tear pattern	–	Horizontal (4/4)	Horizontal (3/7) + Complex (4/7)	–
Position of tibial indentation	–	Posteriorly (4/4)	Central (6/7)	–

**Fig. 2:**
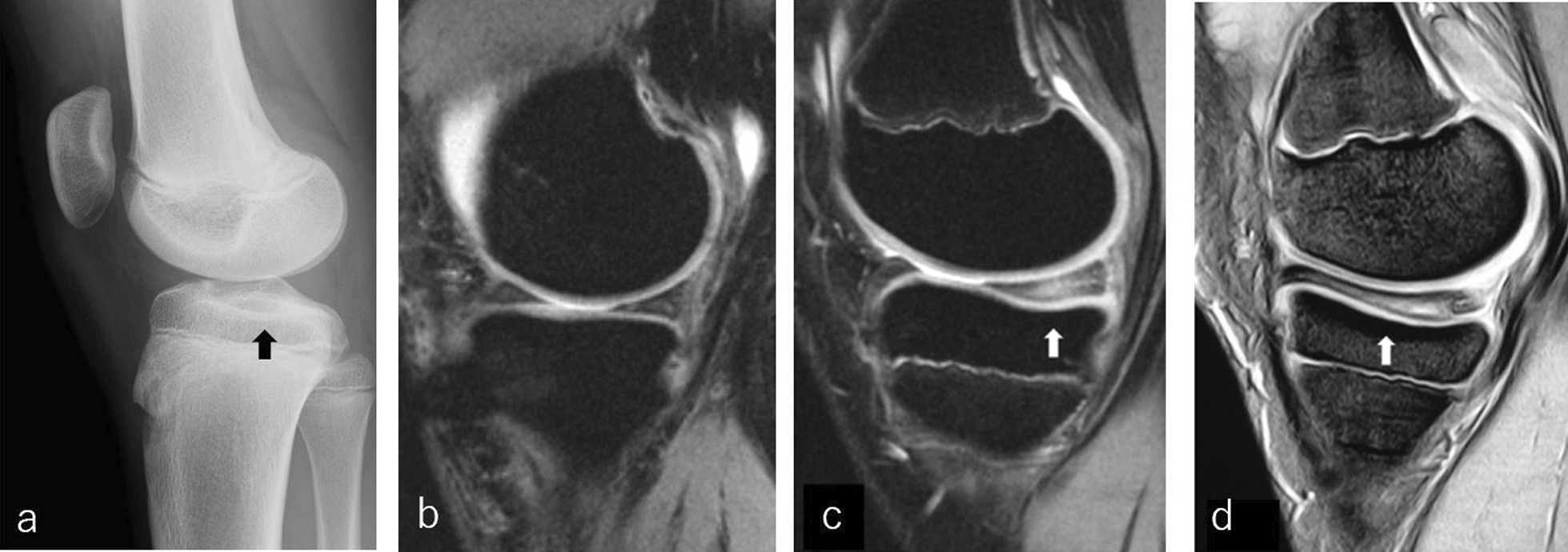
**a** The lateral plain radiograph of a 14-year-old boy; the black arrow indicates the indentation of the medial tibia plateau. **b**–**d** Magnetic resonance sagittal images showing the medial menisci. **b** Normal group (47-year-old man), **c** oversize group (14-year-old boy), **d** discoid group (12-year-old boy)

**Fig. 3:**
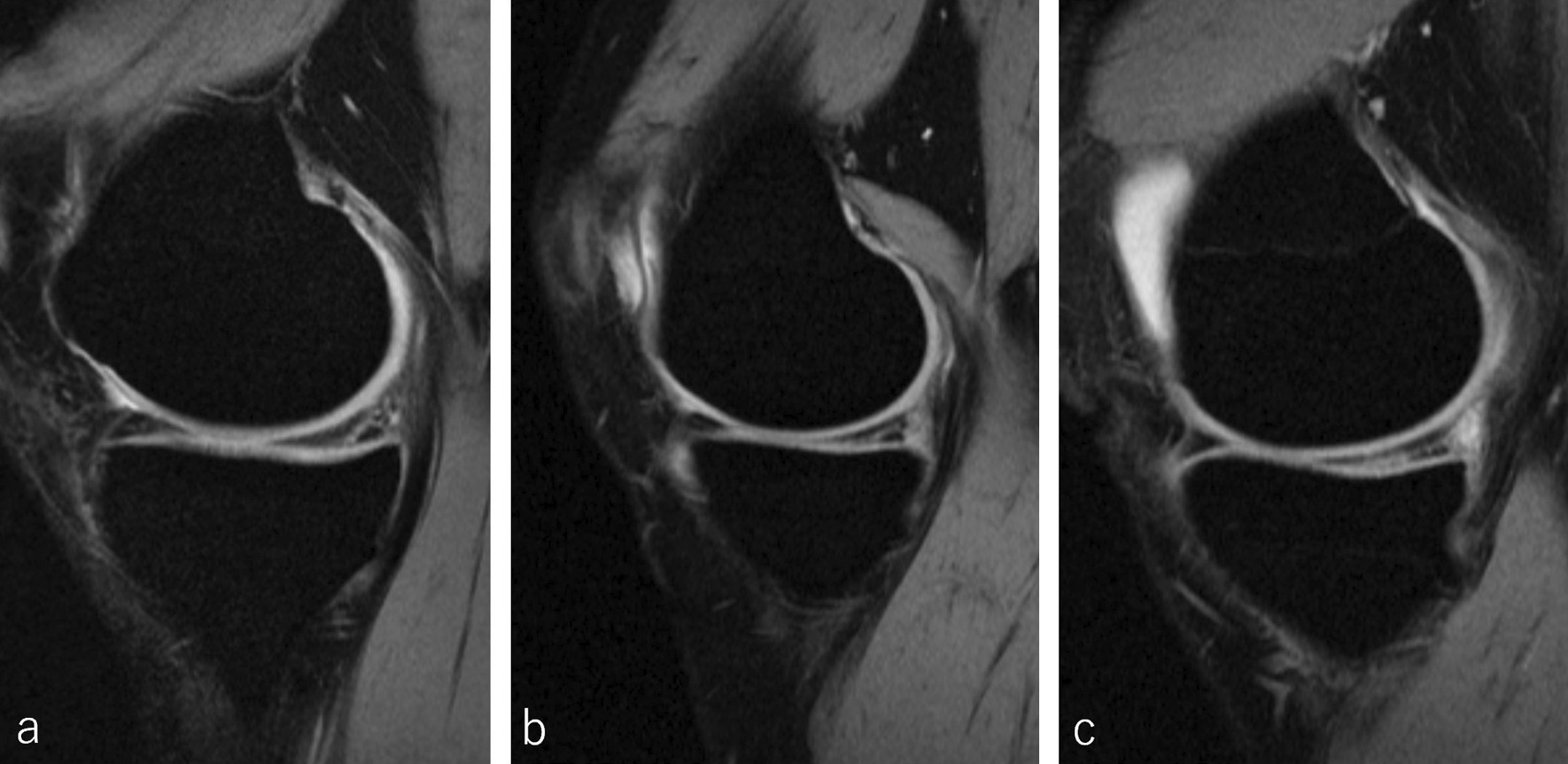
Representative magnetic resonance images of the patients in the oversize group. The sagittal images show the indentation of the medial tibia plateau in **a** a 38-year-old man; **b** a 33-year-old woman; and **c** a 16-year-old boy

Widely spread horizontal tears were observed on MRI in all the patients in the oversize group. On lateral radiographs and sagittal MRI, the indentations of the medial tibial plateau were observed posteriorly in all cases (Figs. 2a, c, 3) (Table 1). Three patients with mild symptoms in the contralateral knee underwent MRI scans, and all patients had a medial meniscus with an oversize posterior segment bilaterally.

**Fig. 4:**
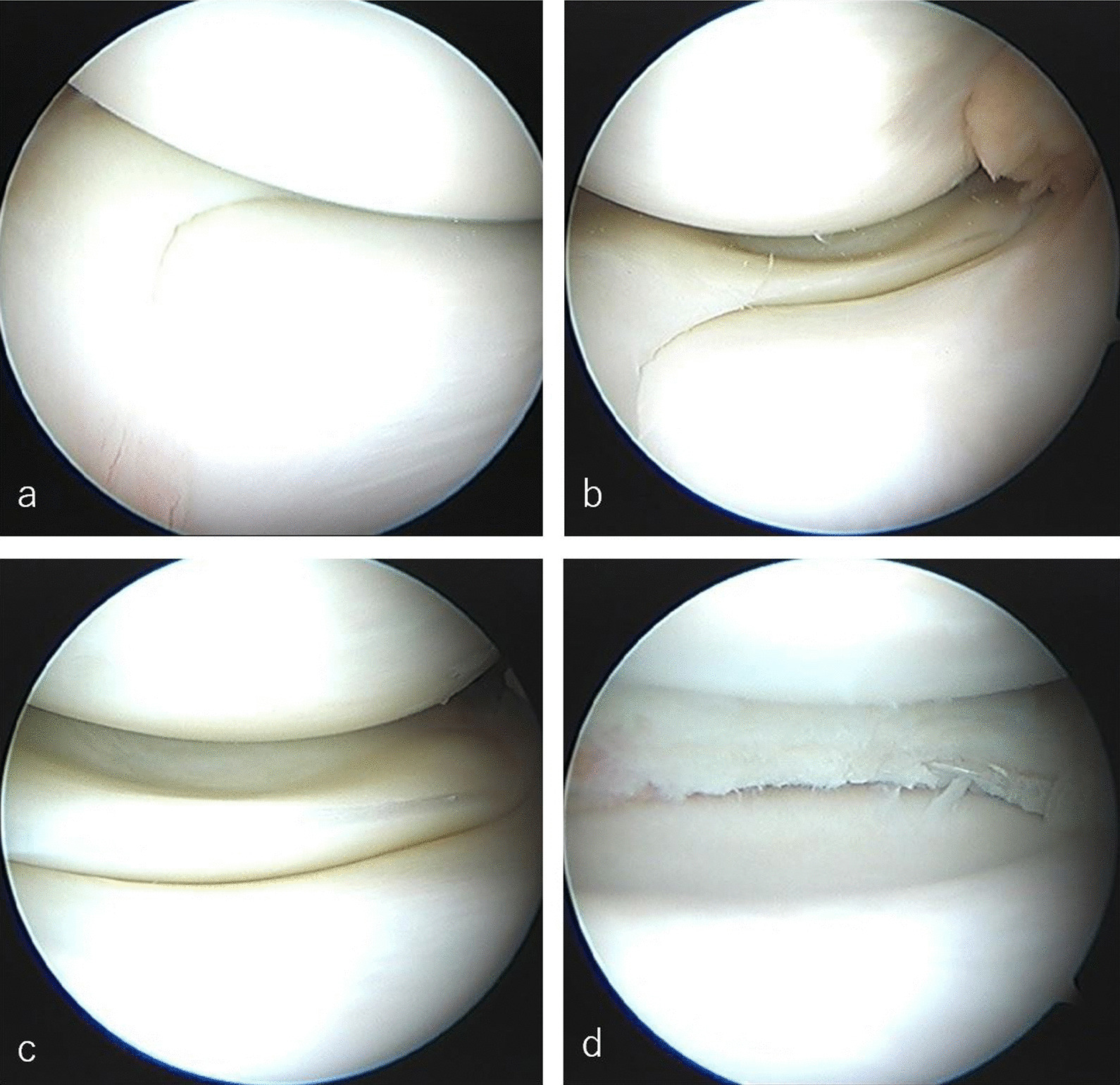
Arthroscopic views of the oversized posterior segment medial meniscus (from the anterolateral portal) of a 14-year-old boy. **a** Normal anterior segment medial meniscus. **b** The mid-body and oversized posterior segment. **c** The oversized posterior segment. **d** Post-partial meniscectomy

**Fig. 5:**
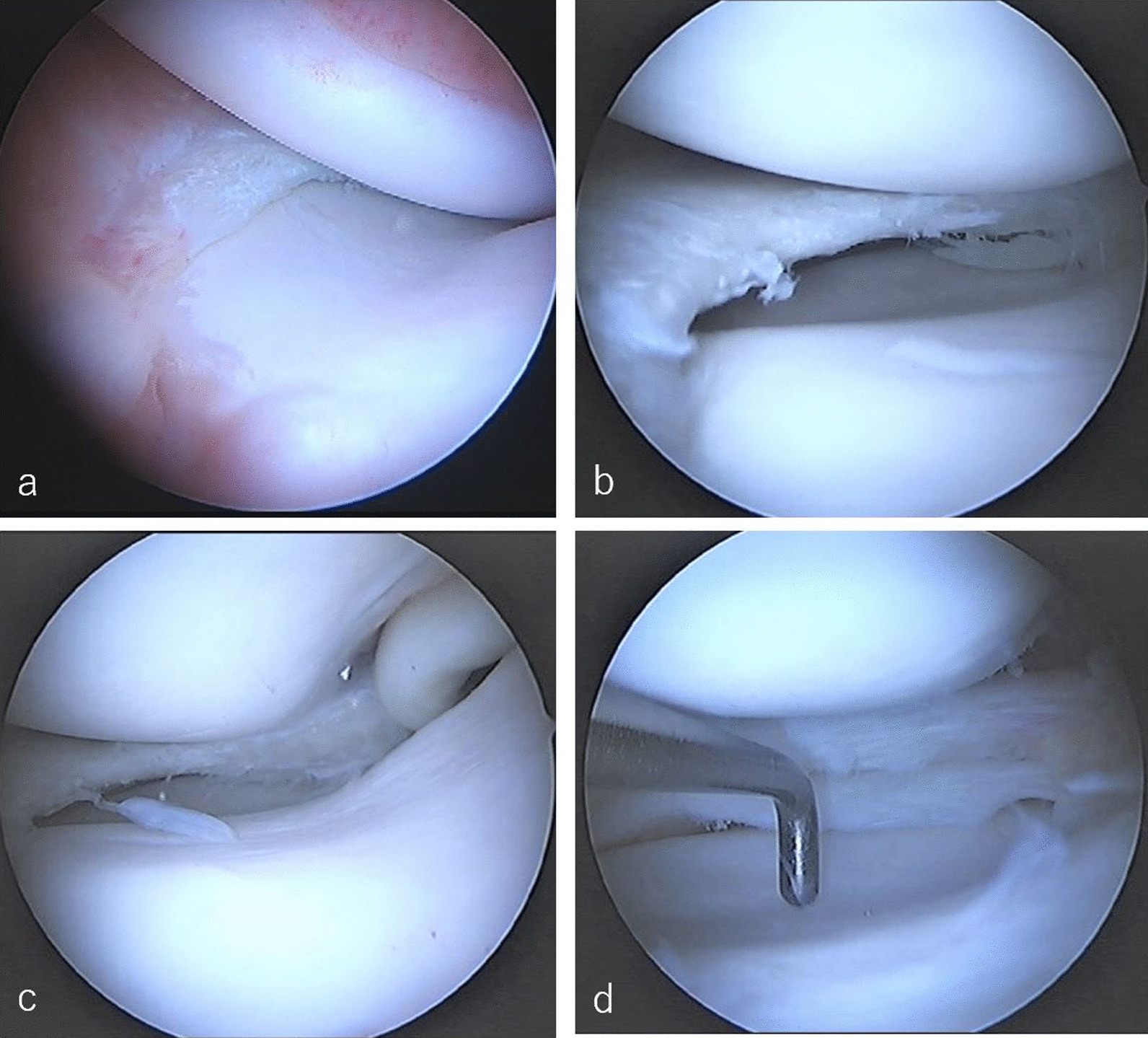
Arthroscopic views of the oversized posterior segment medial meniscus (from the anterolateral portal) of a 16-year-old boy. **a** Normal anterior segment medial meniscus and synovial hyperplasia. **b** The mid-body and oversized posterior segment. **c** Incarceration of the oversized posterior segment medial meniscus in the intercondylar fossa. **d** Post-partial meniscectomy

Arthroscopic examination revealed that the oversized posterior segment covered the posterior half of the medial tibial plateau and the anterior segment appeared normal. Articular surface indentation of the medial tibial plateau was observed in the posterior portion, corresponding to the oversized posterior segment in all cases (Figs. 4, 5). All patients underwent partial meniscectomies. All patients experienced pain relief after the surgery (Figs. 4d, 5d).

### MR measurements

The measurement data for the three groups are listed in Table 2. There was a significant difference in the AP length of the posterior segment between the oversize and normal groups (*P*<0.001). In contrast, no significant differences were observed in the AP lengths of the anterior segment.

**Table 2:** MR measurement

	Normal (*n* = 50)	Oversize (*n* = 4)	Discoid (*n* = 7)	Statistics
AP length of the anterior segment (mm)	9.1 ± 2.1( 4.7–13.6)	9.5 ± 1.9 (8.0–12.2)	N/A	n.s
ML width of the mid-body (mm)	9.3 ± 1.8^a^ (5.9–13.3)	19.9 ± 2.6^b^ (16.1–21.6)	25.8 ± 1.9^c^ (25.5–27.8)	^a, b^, *P* < 0.001 ^b, c^, *P* = 0.010 ^c, a^, *P* < 0.001
AP length of the posterior segment (mm)	14.3 ± 2.8 (8.2–19.3)	23.6 ± 2.8 (20.0–26.5)	N/A	*P* < 0.001
AP length ratio	0.30 ± 0.05 (0.17–0.40)	0.48 ± 0.05 (0.43–0.52)	N/A	*P* < 0.001

Data are presented as mean ± standard deviation (range). *N/A* Not applicable. *n.s.* no statistically significant difference

There were significant differences in the mid-body ML width in the normal, oversize, and discoid groups. The mid-body width of the discoid group was the largest among the three groups. The mid-body ML width in the overweight group was significantly larger than that in the normal group (*P*=0.010).

## Discussion

The most important finding of this study was that we found a new type of morphological abnormality of the medial meniscus. Although there are some reports on the embryology of the discoid meniscus [20–23], this remains largely unclear. Evidence suggests that the meniscus becomes a clearly defined structure by the 8th week of fetal development. The meniscus assumes its relationship with the rest of the knee by the 14th week [24]. Fukazawa et al. reported that the discoid shape is established postnatally due to hypertrophy of the meniscus following lesions caused by detachment of the posterior portion of the meniscus from the tibial plateau [25]. This report presents a new incomplete type of discoid medial meniscus with an oversize posterior segment, which has never been reported previously.

Significantly larger sizes characterized the abnormal morphology of the oversized posterior segment medial meniscus at the mid-body and posterior segment, without a significant difference at the anterior segment compared to the normal group. Previous reports of a discoid medial meniscus pointed out “cupping” [26, 27] of the medial tibial plateau on AP radiographs and the central indentation of the medial tibial plateau on sagittal MRI as characteristic imaging findings [28]. Similar to previous reports, all patients with discoid and oversized posterior segment medial menisci in our study indicated indentation of the medial tibial plateau on lateral radiographs and sagittal MRI. Moreover, we found a difference in the location of the indentation between patients with discoid and oversized posterior menisci. In patients with discoid menisci, indentations were observed near the center of the medial plateau, whereas they were observed in the posterior area in the oversized posterior segment of the medial menisci (Fig. 2b–d).

The medial meniscus is mainly C-shaped, and various shapes of the medial meniscus have been reported, and morphometry of the medial meniscus is also reported [22, 23]. Bloecker et al. reported morphometric differences between the medial and lateral menisci by three-dimensional analysis using MRI [29]. These results were similar in width to the anterior, mid-body, and posterior segments, measured as a normal group at our hospital. In our study, if the AP length of the posterior segment of the medial meniscus exceeded 19.9 mm, the patients were defined as those with an oversized posterior segment. The ML widths of the mid-body were < 14 mm in the normal group, 16–22 mm in the oversize group, and > 25 mm in the discoid group. A significant difference was observed between the normal and oversize groups. In contrast to the posterior and mid-body segments, there were no differences in the anterior segment between the oversize and normal groups (< 14 mm in both groups). Therefore, the oversize posterior segment medial meniscus could be defined with an abnormally oversize posterior segment (≥ 20 mm) and normal anterior segment size (< 14 mm). In addition to the normal size of the anterior segment, the lower ML width of the mid-body could be a possible parameter for distinguishing the oversized posterior segment from the discoid medial meniscus. However, the size of the meniscus is likely to change with sex, age, height, and weight, etc. One report stated that the meniscus size correlated more strongly with the size of the tibial plateau [30]. Therefore, we calculated the AP length ratio of the posterior segment relative to the medial tibial plateau and found a significant difference in the AP length ratio between the oversize group and the normal group (Table 2) (0.48 vs 0.30, *P* < 0.001).

For the classification of discoid lateral menisci, complete, incomplete, and Wrisberg types are often used [7, 31]. Regarding the shape of the discoid lateral meniscus, the central part of the meniscus was smaller and thinner in incomplete discoid lateral menisci than in complete discoid menisci. Notably, medial menisci with oversized posterior segments in our study had larger middle to posterior segments than those with normal medial menisci. The shapes appeared different from the incomplete type, but resembled the Wrisberg-type lateral discoid menisci. Although the oversized posterior segment medial meniscus could be regarded as an “incomplete type of discoid medial meniscus,” we specifically named it as “oversize posterior segment” to clarify the characteristics of this new morphological abnormality in the medial meniscus. Moreover, none of the patients in our study had meniscal anomalies of “abnormal anterior horn insertion on the ACL” [32–34] and “meniscal cyst,” which were reported as coexisting abnormalities with the discoid medial meniscus [35]. Therefore, none of the patients with oversized posterior segment had a discoid medial meniscus characteristic in this study. To the best of our knowledge, there have been no previous reports of incomplete discoid medial menisci and/or oversized posterior segments. Therefore, this report is the first to identify a new type of abnormality of the medial meniscus as an incomplete discoid medial meniscus. We also identified the clinical features of oversized posterior-segment medial menisci. All the patients started experiencing medial knee pain without obvious trauma or strenuous sports, as often observed in patients with discoid lateral and discoid medial menisci [14]. Therefore, if clinicians encounter relatively younger patients who begin having medial pain without any cause and have a wide, severe horizontal tear in the posterior segment, an oversized-posterior-segment medial meniscus can be considered a possible etiology.

This study has some limitations. First, the number of cases with oversized posterior segments was small. As there were four cases, there is a possibility that measurement errors may have increased, and there may be less reliability of the clinical symptoms and imaging findings. Therefore, it is necessary to conduct future studies with a larger number of cases. Second, there is a possibility that there may have been measurement errors because we used MRI for measurements. Thirdly, there is a potential for bias in the measurement of meniscus sizes. Therefore, the measurements were taken twice by two examiners. The intra-class correlation coefficients (ICC) were excellent for all measurements, showing that the measure was reliable (intra: 0.92, inter: 0.90). Finally, although an oversize posterior segment characterized this new type of incomplete discoid medial meniscus as the morphological abnormality, we could not find clearly clinical significancy in this study. However, the incomplete discoid medial meniscus with an oversize posterior segment did not have the morphological features that should be in the discoid. In particular, the incomplete discoid medial meniscus was characterized by the normal anterior segment. We believe that this clearly distinguishes it from the discoid meniscus. Therefore, further research would be required to characterize better the epidemiology of this new type of incomplete discoid meniscus, establish diagnostic methods other than MRI, and provide specific surgical treatment.

## Conclusions

We investigated the clinical characteristics and MRI findings in 11 patients, including 4 with oversized posterior segments and 7 with discoids of the medial menisci. A relatively younger age, subtle symptoms, and sagittal joint surface geometries of the medial tibial plateau were characteristic in these patients. Posterior and center indentations were observed in oversized posterior segment and discoid cases, respectively. An incomplete discoid medial meniscus with an oversized posterior segment meniscus can be defined as a new type of morphological abnormality in the medial meniscus.

## Data Availability

The datasets used and/or analyzed during the current study are available from the corresponding author on reasonable request.

## Abbreviations

MRI: Magnetic resonance imaging
AP: Anteroposterior
ML: Mediolateral
